# Suppression of Wnt Signaling and Osteogenic Changes in Vascular Smooth Muscle Cells by Eicosapentaenoic Acid

**DOI:** 10.3390/nu9080858

**Published:** 2017-08-10

**Authors:** Yukihiro Saito, Kazufumi Nakamura, Daiji Miura, Kei Yunoki, Toru Miyoshi, Masashi Yoshida, Norifumi Kawakita, Tomonari Kimura, Megumi Kondo, Toshihiro Sarashina, Satoshi Akagi, Atsuyuki Watanabe, Nobuhiro Nishii, Hiroshi Morita, Hiroshi Ito

**Affiliations:** 1 Department of Cardiovascular Medicine, Okayama University Graduate School of Medicine, Dentistry, and Pharmaceutical Sciences, Okayama 700-8558, Japan; miura-daiji@nagano-nurs.ac.jp (D.M.); yunokei@yahoo.co.jp (K.Y.); miyoshit@cc.okayama-u.ac.jp (T.M.); masashiyoshid@gmail.com (M.Y.); rm3ta6@bma.biglobe.ne.jp (N.K.); tomonari_ver21@yahoo.co.jp (T.K.); bethnyan.fujituki.sibowchan@gmail.com (M.K.); sarashina.toshihiro@gmail.com (T.S.); akagi-s@cc.okayama-u.ac.jp (S.A.); waatsu2034@yahoo.co.jp (A.W.); nnnnishii2001@gmail.com (N.N.); hmorita@cc.okayama-u.ac.jp (H.M.); itomd@md.okayama-u.ac.jp (H.I.); 2 Department of Basic Medicine, Nagano College of Nursing, Komagane 399-4117, Japan; 3 Department of Chronic Kidney Disease and Cardiovascular Disease, Okayama University Graduate School of Medicine, Dentistry and Pharmaceutical Sciences, Okayama 700-8558, Japan; 4 Department of Cardiovascular Therapeutics, Okayama University Graduate School of Medicine, Dentistry, and Pharmaceutical Sciences, Okayama 700-8558, Japan

**Keywords:** vascular calcification, eicosapentaenoic acid, Wnt signaling

## Abstract

Vascular medial calcification is often observed in patients with arteriosclerosis. It is also associated with systolic hypertension, wide pulse pressure, and fluctuation of blood pressure, which results in cardiovascular events. Eicosapentaenoic acid (EPA) has been shown to suppress vascular calcification in previous animal experiments. We investigated the inhibitory effects of EPA on Wnt signaling, which is one of the important signaling pathways involved in vascular calcification. Intake of food containing 5% EPA resulted in upregulation of the mRNA expression of *Klotho*, an intrinsic inhibitor of Wnt signaling, in the kidneys of wild-type mice. Expression levels of β-catenin, an intracellular signal transducer in the Wnt signaling pathway, were increased in the aortas of *Klotho* mutant (*kl*/*kl*) mice compared to the levels in the aortas of wild-type mice. Wnt3a or BIO, a GSK-3 inhibitor that activates β-catenin signaling, upregulated mRNA levels of *AXIN2* and *LEF1*, Wnt signaling marker genes, and *RUNX2* and *BMP4*, early osteogenic genes, in human aorta smooth muscle cells. EPA suppressed the upregulation of *AXIN2* and *BMP4*. The effect of EPA was cancelled by T0070907, a PPARγ inhibitor. The results suggested that EPA could suppress vascular calcification via the inhibition of Wnt signaling in osteogenic vascular smooth muscle cells via PPARγ activation.

## 1. Introduction

Vascular calcification is observed in arteriosclerosis and reduces aortic and arterial elastance. Intimal calcification is commonly associated with atherosclerosis. Medial calcification is often observed in patients with type 2 diabetes mellitus and end-stage renal disease, and is known as Mönckeberg’s medial sclerosis (MMS). This type of calcification leads to systolic hypertension and fluctuation of blood pressure, finally causing cardiac hypertrophy, myocardial ischemia, peripheral arterial ischemia, and congestive heart failure [1,2,3,4,5].

Eicosapentaenoic acid (EPA) is effective for the prevention and treatment of arteriosclerotic diseases [6,7,8,9]. EPA has also suppressed vascular calcification in animal experiments [10,11,12,13,14]. Since EPA has multiple physiological activities, it is important to clarify the specific mechanism by which vascular calcification is suppressed.

The Wnt signaling pathway is involved in various aging phenotypes, including vascular calcification [15,16,17,18,19,20,21,22]. Patients with chronic kidney disease (CKD) show significant arterial calcification, and have a very high risk of developing cardiovascular diseases [23,24]. They also have mineral and bone disorders, and high phosphate induces vascular calcification through activating Wnt/β-catenin signaling [25]. *Klotho* mutant (*kl*/*kl*) mice show severe tissue calcium deposition, including vascular medial calcification [21]. Since Klotho binds to multiple Wnt ligands and inhibits Wnt signaling activation, Wnt signaling is activated in *kl*/*kl* mice [22,26]. Klotho is mainly produced in the kidneys, and the secreted form is released into the blood [27,28]. Reduced production of klo tho is also observed in Patients with CKD [25].

Since we have observed a remarkable suppressive effect of EPA on arterial calcification of *kl*/*kl* mice [12], we investigated the effect of EPA on Wnt signaling in arterial calcification. We first examined whether EPA can upregulate *Klotho* expression in the kidney. Meanwhile, the intake of powder chow supplemented with 5% purified EPA significantly suppressed vascular calcification in *kl*/*kl* mice, despite a defect of Klotho production [12]. Additionally, whether Klotho is expressed in the artery is controversial [29,30,31]. Thus, we consequently investigated the effects of EPA on Wnt signaling in vascular smooth muscle cells (VSMCs).

## 2. Materials and Methods

### 2.1. Animal Experiment

*Klotho mutant* (*kl*/*kl*) mice were purchased from Clea Japan (Tokyo, Japan). Four-week-old *kl*/*kl* mice and wild-type mice were given diets either containing 5% EPA (Mochida Pharmaceutical Co., Ltd., Tokyo, Japan) (EPA diet), or not containing EPA (control diet), for four weeks. All animal protocols were approved and conducted according to the recommendations of Okayama University on Animal Care and Use. The animal procedures performed conform to the National Institutes of Health (NIH) guidelines (Guide for the Care and Use of Laboratory Animals).

### 2.2. Immunohistochemical Staining

Mice were anesthetized by intraperitoneal injection of 40 mg/kg pentobarbital (Kyoritsu Seiyaku Corporation, Tokyo, Japan). Then, thoracotomy was performed, and the mice were transcardially perfused with saline. The aorta and kidney of each mouse were harvested under a stereoscopic microscope (SZ61, Olympus, Tokyo, Japan). The aorta was embedded in Optimal Cutting Temperature Compound (Sakura Fintek, Tokyo, Japan). The embedded tissues were sectioned at 5 μm in a microtome (CM1850, Leica, Wetzlar, Germany) and mounted on slide glasses (S-0317, Matsunami Glass, Osaka, Japan). They were fixed in 4% paraformaldehyde for 15 min, followed by incubation with Blocking One Histo (Nacalai Tesque, Kyoto, Japan) for 30 min, and then they were stained with primary antibodies against β-catenin (1:100 dilution, D10A8 XP, Cell Signaling Technology, Danvers, MA, USA) and α-SMA (1:400 dilution, Clone 1A4, Sigma Aldrich, St. Louis, MO, USA). The secondary antibodies used were tetramethylrhodamine (TRITC) swine anti-rabbit Ig (1:20, Dako, Santa Clara, CA, USA), and Alexa Fluor 488 goat anti-mouse IgG (1:200, Molecular Probes, Eugene, OR, USA).

### 2.3. Cell Culture

Primary human aortic smooth muscle cells (HAoSMCs) purchased from Lonza (Basel, Switzerland) were cultured in Smooth Muscle Growth Medium-2 (SmGM-2, Lonza). Cells between passages 5–9 were used for all experiments. In loading experiments, cells were cultured in Dulbecco’s Modified Eagle Medium (DMEM) (Invitrogen, Carlsbad, CA, USA) supplemented with 0.5% fetal bovine serum (HyClone, South Logan, UT, USA), and some reagents and were harvested after 48 h. EPA (Sigma Aldrich) was dissolved in ethanol. One hundred ng/mL Wnt3a (R&D Systems, Minneapolis, MN, USA), 1 μg/mL heparin (Sigma Aldrich), 1 μmol/L 6-bromoindirubin-3′-oxime (BIO) (Wako, Osaka, Japan), and/or 10 μmol/L T0070907 (Tocris Bioscience, Bristol, UK) were used in cell culture experiments.

### 2.4. Cell Staining

HAoSMCs were plated on 0.1% gelatin-coated cover glasses, and fixed in 4% paraformaldehyde. The cells were stained with rhodamine-phalloidin (1:200 dilution, Molecular Probes, Eugene, OR, USA) and Hoechst 33342 (1:5000 dilution, Molecular Probes). Rhodamine-phalloidin is an F-actin probe conjugated to the red-orange fluorescent dye, tetramethylrhodamine. Hoechst 33342 is a blue dye for cell fluorescence of nuclei.

### 2.5. Quantitative PCR

Murine kidneys in Trizol Reagent were homogenized using Bead Crusher μT-01 (Taitec, Saitama, Japan). HAoSMCs were also lysed using Trizol Reagent. Total RNA was extracted using a Trizol Plus RNA Purification Kit (Invitrogen, Carlsbad, CA, USA). Complementary DNA was synthesized from less than 1 μg of total RNA using a SuperScript VILO cDNA Synthesis Kit (Invitrogen), as prescribed in the manual and subjected to PCR amplification. Taq DNA polymerase (Takara, Shiga, Japan) was used for reverse transcription-polymerase chain reaction (RT-PCR), and PCR products were subjected to electrophoresis in 2% agarose gels and stained with ethidium bromide. KAPA SYBR Fast qPCR Kit (Kapa Biosystems, Wilmington, MA, USA) and Applied Biosystems 7300 Real-Time PCR Systems (Applied Biosystems, Foster City, CA, USA) were used for quantitative PCR (*q*-PCR). The *q*-PCR data were processed by a *ΔΔ*CT method. PCR primers are shown in Table 1. The *q*-PCR experiments were performed in triplicate four times.

### 2.6. FFAR4 Knockdown by siRNA

To knockdown *FFAR4* in HAoSMCs, 5 μmol/L small interfering RNA (siRNA, s200889, Ambion, Foster City, CA, USA) was transfected using Lipofectamine RNAiMAX (Invitrogen) according to the manufacturer’s instructions. *FFAR4* downregulation was confirmed by RT-PCR.

### 2.7. Statistical Analysis

All data are expressed as means ± SE. Statistical analysis was performed by Student’s *t* test for unpaired data or one-way ANOVA, with comparison of different groups by Tukey’s post hoc test using SPSS statistics 24 (IBM, Armonk, NY, USA). Values of *p* < 0.05 were considered to be significant.

## 3. Results

### 3.1. Intake of EPA Food Upregulated Klotho Expression in Kidneys of Wild-Type Mice

**Figure 1:**
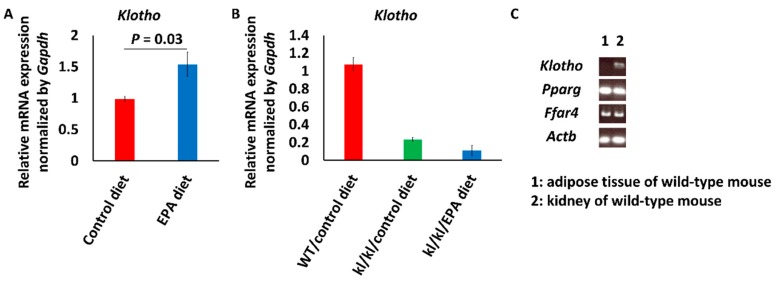
*Klotho* (*kl*/*kl*) mRNA expression levels in kidneys of mice. (**A**) *Klotho* mRNA expression levels in kidneys of wild-type mice fed a diet with or without eicosapentaenoic acid (EPA). Data are shown as means ± SE (Control diet, *n* = 4; EPA diet, *n* = 8) and were analyzed using Student’s *t* test; (**B**) *Klotho* mRNA expression levels in kidneys of *kl*/*kl* mice fed a diet with or without EPA. Data are shown as means (*n* = 2 in each group); (**C**) Reverse transcription-polymerase chain reaction (RT-PCR) showed the mRNA of EPA receptors, *Pparg* and *Ffar4*, in the kidney.

*Klotho* mRNA levels in mice fed the EPA diet (*n* = 8) were about 1.5 times higher than the levels in mice fed the control diet (*n* = 4) (Figure 1A). In *kl*/*kl* mice, the intake of EPA did not change serum phosphate, and did not increase *Klotho* expression (control diet, *n* = 2; EPA diet, *n* = 2) (Figure 1B) [12]. Expression of the nuclear receptor and plasma membrane receptor of EPA, PPARγ and FFAR4, respectively, were confirmed by RT-PCR (Figure 1C).

### 3.2. Beta-Catenin Expression Increased in the Aortas of Kl/kl Mice

**Figure 2:**
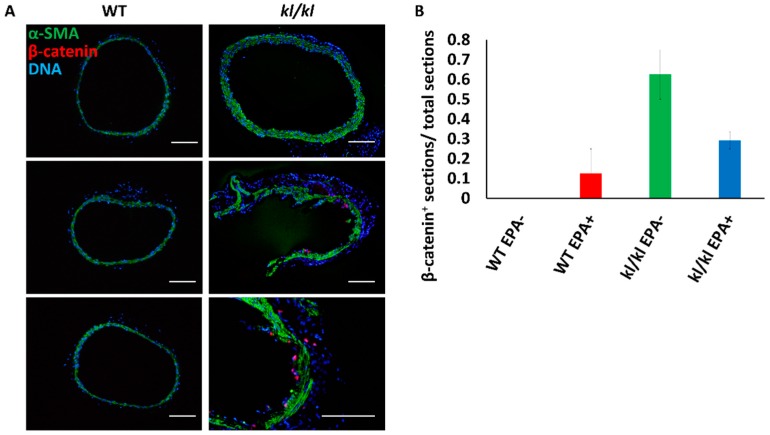
Beta-catenin expression in aortas of mice. (**A**) Immunostaining of mouse aortas: green, α-smooth muscle actin (α-SMA); red, β-catenin; and blue, DNA; (**B**) Proportions of β-catenin-positive aorta sections (total numbers of sections: Wild-type (WT) mice fed control diet, *n* = 7; WT mice fed EPA diet, *n* = 6; *kl*/*kl* mice fed control diet, *n* = 6; and *kl*/*kl* mice fed EPA diet, *n* = 7). Bar = 100 μm.

The medial layer, which was α-SMA positive, showed hyperplasia, while the expression of β-catenin, an intracellular signal transducer in the Wnt signaling pathway, was detected in more sections of aortas from *kl*/*kl* mice (control diet, number of animals = 2 and number of tissue sections = 7; EPA diet, number of animals = 2 and number of tissue sections = 6) than in sections of aortas from wild-type mice (control diet, number of animals = 2 and number of tissue sections = 6; EPA diet, number of animals = 2 and number of tissue sections = 7) (Figure 2A,B).

### 3.3. EPA Suppressed the Expression of Wnt Signaling Marker and Early Osteogenic Genes

**Figure 3:**
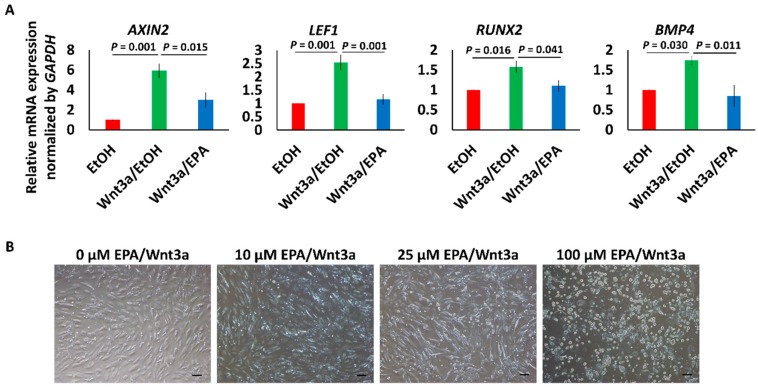
Effects of Wnt3a and EPA on human aortic smooth muscle cells (HAoSMCs). (**A**) Changes of the gene expression pattern in HAoSMCs treated with or not treated with 100 ng/mL Wnt3a and/or EPA. Data are shown as means ± SE (*n* = 4 in each group), and were analyzed using one-way ANOVA with Tukey’s post hoc test. EtOH means ethanol; (**B**) Cytotoxicity of EPA in HAoSMCs. Bar = 100 μm.

Wnt3a (100 ng/mL) increased not only the expression of Wnt signaling marker genes *AXIN2* and *LEF1*, but also the expression of *RUNX2* and *BMP4*, which are genes associated with vascular calcification [20,32,33,34,35]. Upregulation of these genes was suppressed by 25 μmol/L EPA (Figure 3A). EPA did not completely suppress the upregulation genes by Wnt3a at 10 μmol/L, and 100 μmol/L EPA induced cell death (Figure 3B). We therefore used 25 μmol/L EPA in subsequent experiments.

### 3.4. EPA Suppressed β-Catenin Signaling via PPARγ Activation

**Figure 4:**
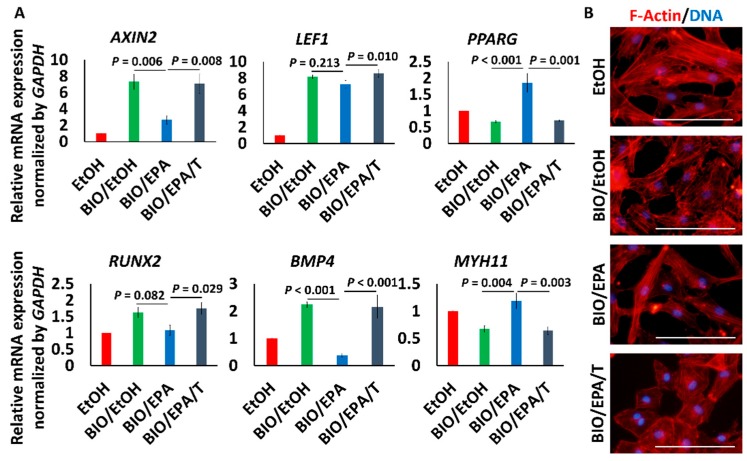
Effects of EPA on β-catenin signaling stimulated by GSK-3 inhibitor. (**A**) Changes of the gene expression pattern in HAoSMCs treated with 1 μmol/L BIO, with or without EPA/T0070907. Data are shown as means ± SE (*n* = 4 in each group) and were analyzed using one-way ANOVA with Tukey’s post hoc test. T means T0070907 (10 μmol/L); (**B**) Morphological changes of HAoSMCs. Red and blue represent *f*-actin and DNA, respectively. Bar = 100 μm.

**Figure 5:**
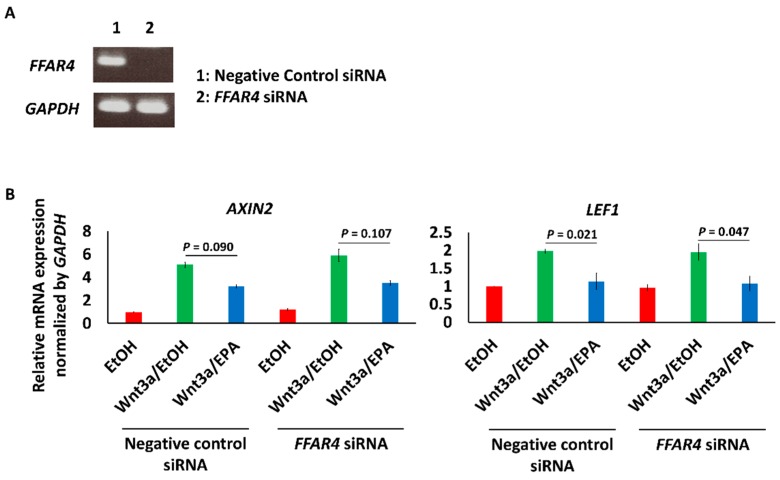
Effect of the knockdown of *FFAR4* on Wnt signaling in HAoSMCs. (**A**) The knockdown of *FFAR4* in HAoSMCs was confirmed by RT-PCR; (**B**) The effect of knockdown of *FFAR4* on the expression of *AXIN2* and *LEF1*, downstream genes of Wnt signaling. Data are shown as means ± SE (*n* = 3 in each group) and were analyzed using one-way ANOVA with Tukey’s post hoc test.

BIO (1 μmol/L), a GSK-3 inhibitor that activates β-catenin signaling, also increased the expression of *AXIN2*, *LEF1*, *RUNX2* and *BMP4*. Upregulation of *AXIN2* and *BMP4* was significantly suppressed, and *RUNX2* tended to be suppressed by 25 μmol/L EPA. EPA is known as a PPARγ agonist and also increased *PPARG* expression. The effects of EPA were cancelled by 10 μmol/L T0070907, a PPARγ inhibitor (Figure 4A). Additionally, BIO made actin fibers sparse, and EPA modestly mitigated the morphological change. The expression of *MYH11*, a mature contractile smooth muscle cell marker, was decreased by BIO, and the expression was recovered by EPA. T0070907 cancelled the effect of EPA (Figure 4A,B). The knockdown of *FFAR4*, a receptor of ω-3 fatty acids, did not cancel the inhibitory effect of EPA on *AXIN2* and *LEF1* expression (Figure 5A,B) [36].

## 4. Discussion

**Figure 6:**
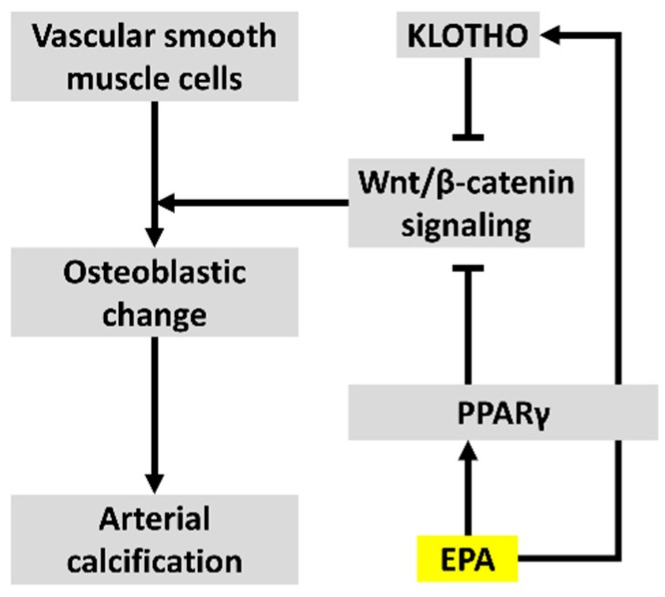
Mechanisms by which EPA suppresses vascular calcification via inhibition of Wnt signaling.

Our study revealed that EPA upregulated the expression of *Klotho*, a Wnt signaling inhibitor, in the kidney and inhibited Wnt signaling in osteogenic VSMCs via PPARγ, as shown in Figure 6. These results suggested that the effects could lead to the suppression of vascular calcification. In addition to our previous study, the results of the present study using human VSMCs suggested that EPA can also affect human vascular calcification by the suppression of Wnt signaling [12]. This study is therefore a step toward the pathology of arteriosclerosis.

The intake of EPA increased *Klotho* mRNA expression in the kidney, as shown in Figure 1. Klotho inhibits the binding of Wnt ligands to their receptors [26]. The upregulated expression of Klotho in the kidney is presumed to suppress vascular calcification; however, we did not examine how EPA upregulated Klotho expression. Meanwhile, PPARγ activation has been reported to be one of the methods for increasing Klotho production in the kidney, because the *Klotho* gene has a PPAR-responsive element (PPRE) [25,37]. Additionally, EPA can directly activate PPARγ [38]. The upregulation of expression by EPA that was observed in our study is a compatible result. However, wild-type mice were used in this study to examine *Klotho* expression in the kidneys. Thus, it is unclear whether EPA increases Klotho production in a pathological situation. Further investigation using other pathological model animals without a Klotho defect is therefore required.

We previously reported that intake of an EPA diet also significantly suppressed vascular calcification in *kl*/*kl* mice, despite a defect of Klotho production [12]. Furthermore, it is not clear whether vascular Klotho is expressed endogenously [29,30,31]. Thus, we consequently investigated the direct effects of EPA on Wnt signaling in the aorta. Increased β-catenin expression in the aortas of *kl*/*kl* mice, as shown in Figure 2, suggested an enhancement of Wnt/β-catenin signaling in the calcified aorta.

Vascular calcification in the tunica media observed in *kl*/*kl* mice and CKD patients is a characteristic manifestation [21,39]. We therefore investigated the effect of EPA in VSMCs, which are a component of the tunica media. We revealed that the enhancement of Wnt/β-catenin signaling by a Wnt ligand or a GSK-3 β inhibitor resulted in osteogenic changes of human VSMCs, as previous studies have demonstrated [18,19,20,34,40]. Furthermore, EPA suppressed the changes, as shown in Figure 3 and Figure 4. EPA has also been reported to prevent palmitic acid-induced osteoblastic changes in VSMCs [41]. The mechanisms by which EPA suppresses vascular calcification have been reported to be a reduction of matrix metalloproteinase production, and a reduction of oxidative stress [11,12]. It has been shown that ω-3 fatty acids are known to suppress Wnt signaling in various types of cancer cells [42,43,44]. Thus, our results, shown in Figure 4, suggested that similar mechanisms might act in osteogenic VSMCs via activation of PPARγ.

We previously reported that FFAR4 signaling is responsible for decreasing oxidative stress in mice [12]; however, FFAR4 was not associated with the inhibitory effects of EPA on Wnt signaling in this experimental situation, as shown in Figure 5. These results suggested that FFAR4 signaling might suppress vascular calcification by other mechanisms.

It is not known whether a decrease in coronary calcification reduces cardiovascular events. Vascular calcification seems to prevent the rupture of vulnerable plaques, because a statin could increase vascular calcification, despite the reduction of cardiovascular events [45,46]. Evaluation using optical coherence tomography and intravascular ultrasound demonstrated that adding on EPA to statin did not change calcified plaque volume; however, it did stabilize the plaques of human coronary arteries [47,48]. Our study revealed that EPA suppresses Wnt signaling in smooth muscle cells. EPA is also known to affect macrophages and endothelial cells [49,50,51]. Additionally, Wnt signaling is associated with inflammation and aging [52,53]. EPA therefore seems to affect not only vascular calcificationm but also other arteriosclerotic processes via the modulation of Wnt signaling [54].

Since the amount of intake of EPA in this study is extremely large for humans, we should look for a specific target for clinical implication. EPA is converted to metabolites with various physiological activities in vivo [55,56,57,58]. We did not examine metabolites in this study because it is difficult to understand the effects of metabolites produced by cells other than VSMCs, e.g., endothelial cells, in an in vitro study [59]. Thus, analyses of metabolites in each tissue or organ might be useful to gain a deeper understanding of the effects of EPA on vascular calcification.

In conclusion, this study revealed that EPA increased the expression of Klotho, an inhibitor of Wnt signaling, in the kidney, and inhibited osteogenic change via enhancement of Wnt signaling in VSMCs. These effects of EPA might lead to suppression of vascular calcification.

## Figures and Tables

**Table 1:** PCR primer pairs.

		Sequence	Annealing Temperature (°C)	Product Size (bp)
Mouse				
*Klotho*	forward	CAAAGTCTTCGGCCTTGTTC	60	111
	reverse	CTCCCCAAGCAAAGTCACA		
*Pparg*	forward	ATCATCTACACGATGCTGGCC	60	81
	reverse	CTCCCTGGTCATGAATCCTTG		
*Ffar4*	forward	TGCCCCTCTGCATCTTGTTC	60	202
	reverse	CGCGATGCTTTCGTGATCTG		
*Actb*	forward	GGAGGGGGTTGAGGTGTT	60	70
	reverse	GTGTGCACTTTTATTGGTCTCAA		
Human				
*AXIN2*	forward	AGTGTGAGGTCCACGGAAAC	58	103
	reverse	CTGGTGCAAAGACATAGCCA		
*LEF1*	forward	AATGAGAGCGAATGTCGTTGC	60	137
	reverse	GCTGTCTTTCTTTCCGTGCTA		
*RUNX2*	forward	TCTTAGAACAAATTCTGCCCTTT	58	136
	reverse	TGCTTTGGTCTTGAAATCACA		
*BMP4*	forward	GATCCACAGCACTGGTCTTG	60	150
	reverse	GGGATGCTGCTGAGGTTAAA		
*PPARG*	forward	GGCTTCATGACAAGGGAGTTTC	60	74
	reverse	AACTCAAACTTGGGCTCCATAAAG		
*MYH11*	forward	AGATGGTTCTGAGGAGGAAACG	60	85
	reverse	AAAACTGTAGAAAGTTGCTTATTCACT		
*FFAR4*	forward	CTGTGCAGGAATGAGTGGAAG	60	197
	reverse	CTGATGGAGGGTACTGGAAATG		
*GAPDH*	forward	GCGAGATCCCTCCAAAATCAA	58	172
	reverse	GTTCACACCCATGACGAACAT		
